# Primary Signet Ring Cell Carcinoma of the Lung: A Rare Histologic Variant With Distinct Radiologic Presentation

**DOI:** 10.7759/cureus.92069

**Published:** 2025-09-11

**Authors:** Najlae Demnati Sadki, Ouiame El Meliani, Hind Majd, Mohammed Tarik Saoudi, Kaoutar Maadin, Abdelilah Khallaf, Lamiae Amaadour, Karima Oualla, Zineb Benbrahim, Samia Arifi, Nawfel Mellas, Mustapha Maaroufi

**Affiliations:** 1 Medical Oncology, Hassan II University Hospital of Fez, Fez, MAR; 2 Diagnostic Radiology, Hassan II University Hospital of Fez, Fez, MAR

**Keywords:** cancer prognosis, chemotherapy response, lung cancer with gastrointestinal features, primary metastatic lung tumor, signet-ring cell carcinoma

## Abstract

Signet ring cell carcinoma (SRCC) is a rare and distinct subtype of adenocarcinoma. It can arise in various organs, including the stomach, colon, breast, bladder, and prostate. It may also occur as a primary pulmonary tumor.

We report a case of a 65-year-old woman with no significant medical history, who presented with a two-month history of dyspnea and hemoptysis, associated with weight loss. A chest CT scan revealed a locally advanced pulmonary mass with mediastinal lymphadenopathy and bilateral pulmonary metastases. A biopsy was performed and confirmed a diagnosis of pulmonary-origin signet ring cell adenocarcinoma.

Given the patient’s general condition, chemotherapy with weekly paclitaxel and carboplatin (AUC2) was initiated. At the first evaluation after three cycles, the disease was stable. The patient subsequently developed a respiratory infection due to a multidrug-resistant organism, and despite appropriate antibiotic therapy, she passed away because of the infection.

Primary SRCC of the lung remains a diagnostic and therapeutic challenge, with poor prognosis despite early intervention. The rapid clinical decline observed in this case highlights the aggressive nature of the disease and the importance of supportive care.

## Introduction

Primary lung signet ring cell carcinomas (SRCCs) are extremely rare, with only a few cases reported; however, they do occur and were first described as a distinct disease entity with specific clinicopathological features and poor prognosis by Kish et al. in 1989 [1]. SRCC was subsequently incorporated into the World Health Organization (WHO) classification as a histological subtype of adenocarcinoma.

This article aims to illustrate the radiologic presentation of primary lung SRCC in correlation with histopathologic and immunohistochemical findings, and to underscore the role of multimodality imaging in accurate diagnosis and clinical management.

## Case presentation

We report a case of a 65-year-old female patient with no significant past medical history, notably with no active or passive smoking history. She presented with New York Heart Association (NYHA) stage II dyspnea, associated with a productive cough of mucoid sputum and intermittent, low-volume hemoptysis, without other respiratory or systemic symptoms. These symptoms had been evolving over a two-month period in the context of general health deterioration.

**Figure 1:**
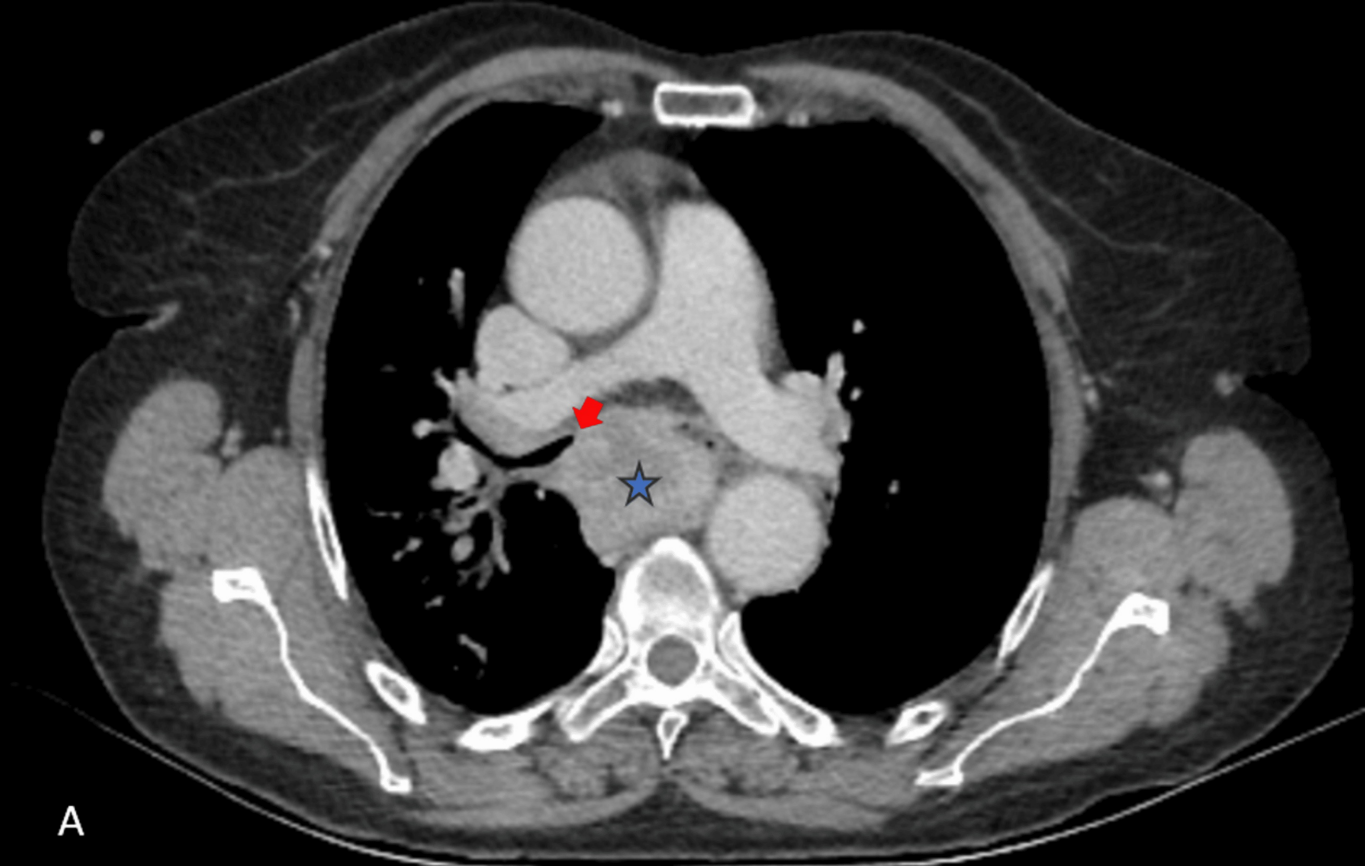
Axial contrast-enhanced CT showing a subcarinal mediastinal mass (blue star) invading the carina (red arrow).

**Figure 2:**
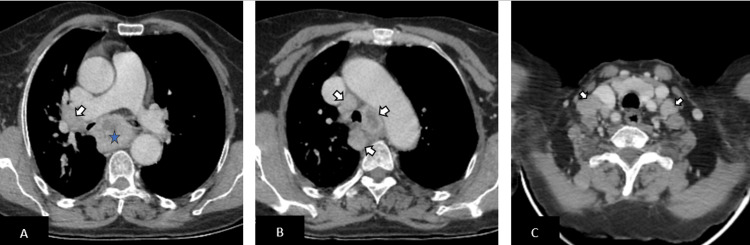
Baseline axial contrast-enhanced CT images (A, B, and C) revealed enlarged lymph nodes in the right hilar, pretracheal, paratracheal, and supraclavicular (arrows), consistent with multifocal mediastinal and cervical nodal involvement.

**Figure 3:**
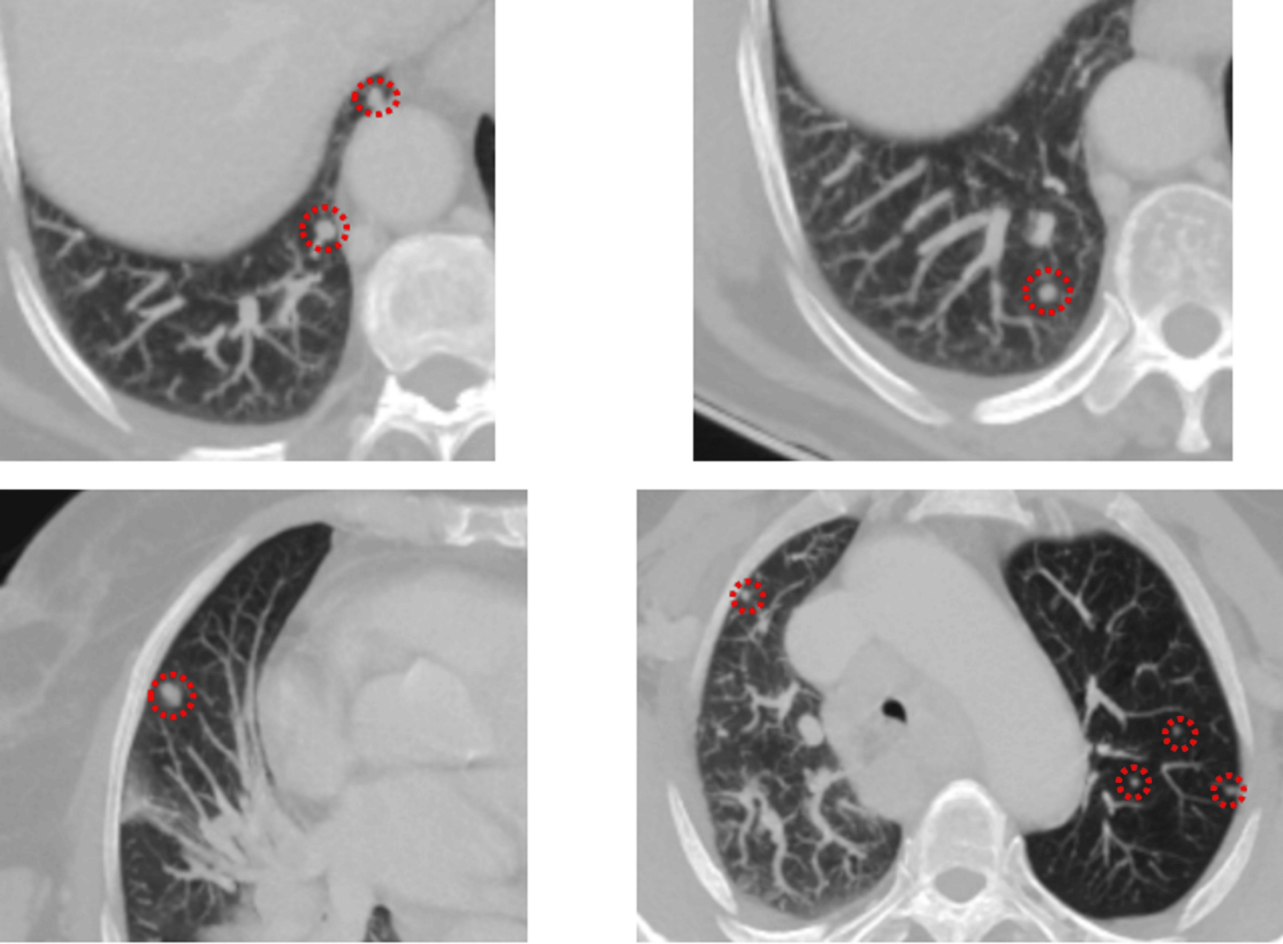
Axial and magnified CT images showing multiple bilateral pulmonary parenchymal nodules (circles), consistent with secondary metastatic lesions.

An initial chest CT scan revealed a locally advanced pulmonary mass associated with multiple mediastinal lymphadenopathies and bilateral pulmonary micronodules and nodules (Figures 1-3).

Bronchoscopic evaluation showed near-complete stenosis of both main bronchi due to an infiltrative and exophytic lesion involving the carina. Exploration of the distal bronchi was difficult but showed no visible lesions. The stenosis was firm and bled easily upon contact. Histopathological analysis revealed a clear cell tumor proliferation. Immunohistochemistry showed positivity for CK7 and TTF1, and negativity for CK20, P40, PAX8, and CDX2. Alcian blue special staining was positive, supporting the diagnosis of a signet ring cell adenocarcinoma.

Staging with a thoraco-abdomino-pelvic CT scan showed a 6.7 cm carinal soft-tissue mass involving both main bronchi with significant narrowing of the bronchial lumen, in intimate contact with the esophagus and surrounding vascular structures. It also revealed bilateral pulmonary nodules, supradiaphragmatic lymphadenopathies, and a heterogeneous soft tissue infiltration of the left lumbar ureter, associated with moderate upstream hydronephrosis.

The case was discussed at a multidisciplinary tumor board. A diagnosis of metastatic signet ring cell adenocarcinoma of pulmonary origin was retained. Testing for EGFR and ALK mutations was negative. A colonoscopy and upper endoscopy were requested to rule out extrapulmonary origins, but were not performed due to the patient's refusal.

Initial blood work revealed renal impairment secondary to ureterohydronephrosis, with a serum creatinine of 16 mg/L and a creatinine clearance (Cockcroft) of 32 mL/min. Urology was consulted for further management.

Given the patient's general condition, she was started on weekly paclitaxel combined with carboplatin (AUC2).

**Figure 4:**
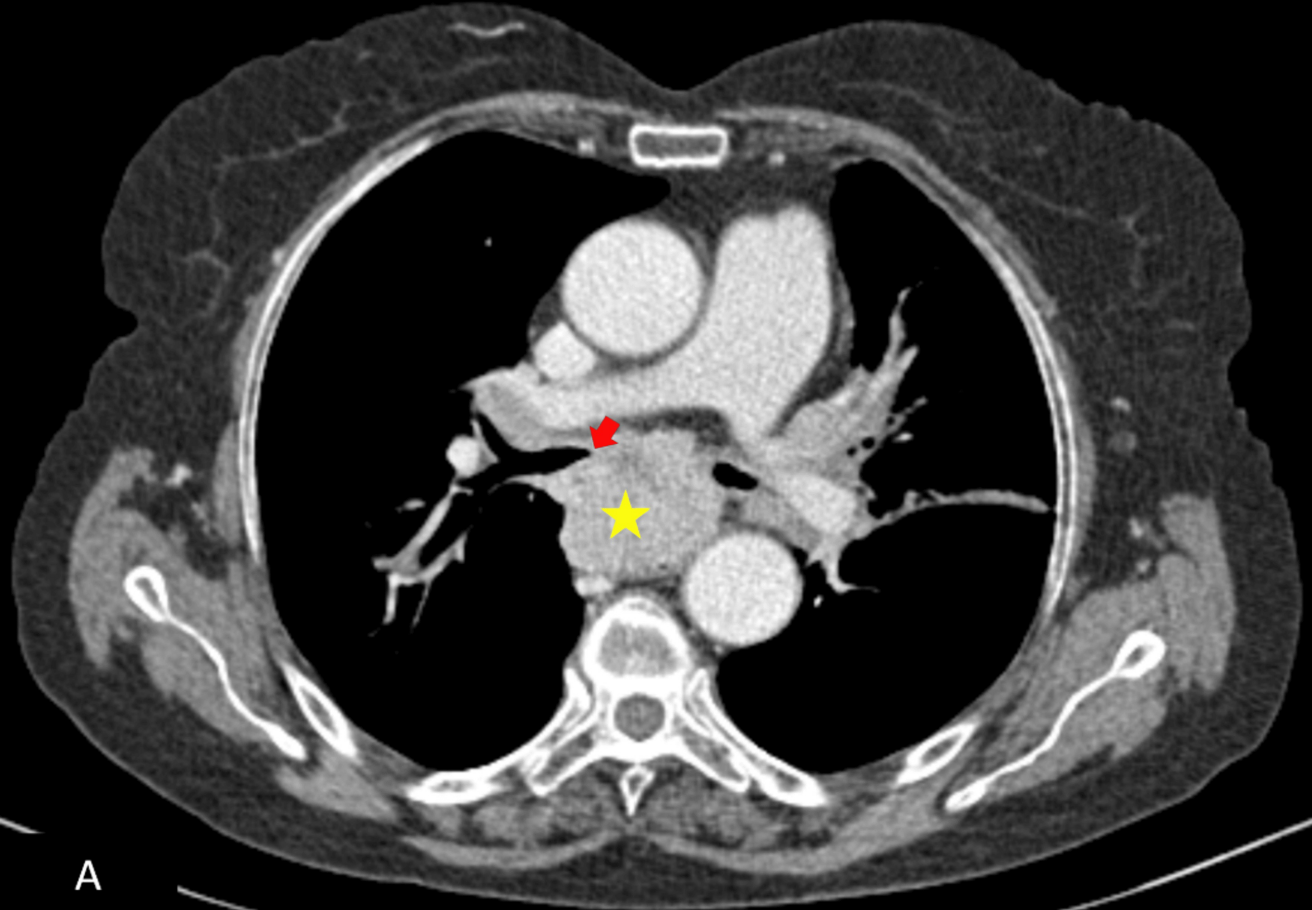
Control imaging after chemotherapy shows a sensitive stability in the volume of the subcarinal mediastinal mass (yellow star), invading the carina (red arrow) and exhibiting intimate contact with the aortic arch over more than 90° of its circumference.

**Figure 5:**
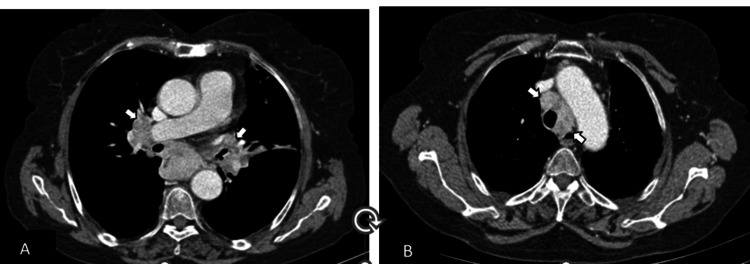
Follow-up post-chemotherapy imaging demonstrates a mild reduction in mediastinal and supraclavicular lymphadenopathy, with the development of central necrosis in some nodes, suggestive of a therapeutic response.

**Figure 6:**
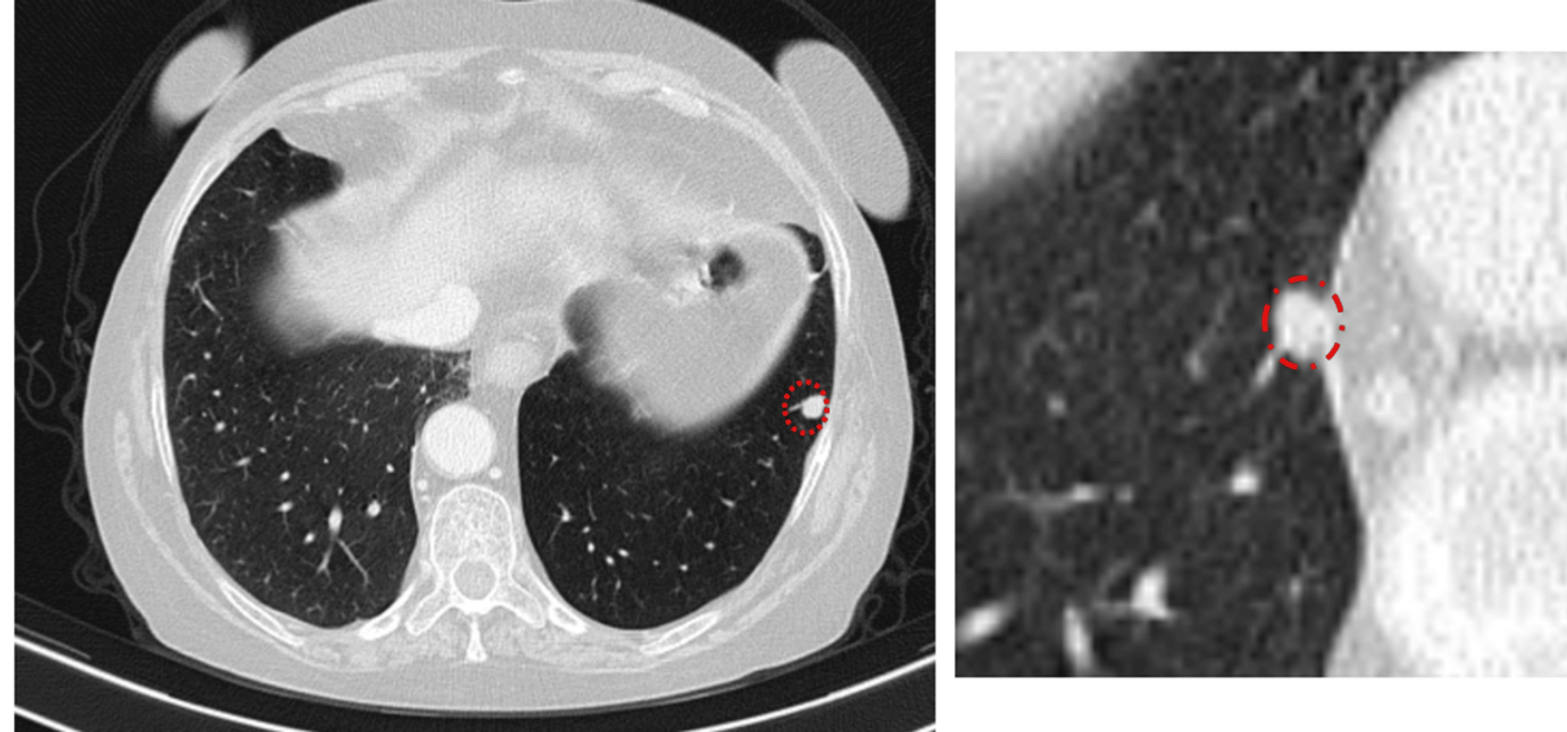
Post-chemotherapy axial and magnified CT images demonstrating a reduction in the number of pulmonary nodules and micronodules, consistent with a partial therapeutic response.

After three cycles, the first follow-up CT scan showed a stable appearance of the mediastinal tumor and metastatic lesions (Figures 4-6). However, new findings included a thrombosis of a segmental branch of the right lower lobe pulmonary artery, extending to subsegmental branches, as well as a left renal vein thrombosis reaching the ipsilateral ovarian vein. Anticoagulant therapy was initiated.

Given the disease stability, the same chemotherapy protocol was continued. Unfortunately, the patient developed a respiratory infection caused by a multidrug-resistant organism, and despite appropriate antibiotic treatment, she died from infectious complications. The time between diagnosis and death was six months.

## Discussion

Differentiating primary pulmonary signet ring cell carcinomas (LSRCCs) from metastatic lesions is essential for guiding appropriate patient management, as their treatment strategies and prognoses differ significantly. Given the exceptional rarity of LSRCC, most of the existing data stems from individual case reports or limited case series. In a 2018 study, Wu et al. analyzed a cohort of 738 patients diagnosed with primary pulmonary SRCC. Their findings revealed that the majority of patients presented with poorly differentiated tumors and distant metastases at the time of diagnosis. Furthermore, these patients were less likely to receive aggressive therapeutic interventions. The reported five-year cancer-specific survival (CSS) rate was merely 11%, with a median CSS of just six months, underscoring the generally poor prognosis associated with this rare histological subtype of lung cancer [2].

Accurate diagnosis of LSRCC requires both morphological evaluation and immunohistochemical (IHC) profiling, particularly to exclude metastases from extrapulmonary sites such as the gastrointestinal tract, breast, or prostate. In their analysis of IHC features, Merchant et al. reported that thyroid transcription factor-1 (TTF-1) is highly expressed in primary pulmonary SRCC, whereas it is typically absent in SRCCs originating from other organs. Additionally, a CK7-positive/CK20-negative profile was observed in 94.1% of primary lung SRCC cases. These markers (TTF-1, CK7, and CK20) are therefore considered valuable tools in the differential diagnosis of primary pulmonary SRCC. [3] Given that the lungs are a frequent site of metastasis, it is essential to exclude an extrapulmonary primary origin before confirming a lung primary.

Clinically, patients typically present at an advanced stage with mediastinal invasion, bilateral pulmonary nodules, and lymphadenopathy, as in our case. Studies by Tsuta et al. [4] confirm that LSRCC is more likely to present with extensive nodal involvement and distant metastases compared to other histological subtypes. In our case, the imaging findings revealed bilateral pulmonary nodules with mediastinal lymphadenopathy and pleural effusion, consistent with the advanced stage at which LSRCC typically presents. Previous reports have demonstrated that LSRCC frequently manifests on computed tomography (CT) as solid, peripherally located nodules, often with irregular or spiculated margins, lobulation, and pleural retraction, reflecting its aggressive nature. Mediastinal lymphadenopathy and pleural effusion, as seen in our patient, are common findings and often indicate nodal metastasis and pleural invasion, which contribute to the poor prognosis associated with this entity. Additionally, the absence of a central mass and the tendency for peripheral distribution may lead to diagnostic delays, particularly when symptoms are nonspecific or the disease is detected incidentally.

Unlike typical lung adenocarcinomas, which may display air bronchograms, cavitation, or central necrosis, LSRCC rarely shows such features, which may serve as a diagnostic clue for radiologists. A retrospective analysis by Yang et al. noted that most LSRCC lesions appear as homogeneous solid nodules without cavitation, and frequently exhibit pleural indentation and adjacent bronchovascular bundle involvement, suggesting early lymphovascular spread [5]. In this report, however, the tumor shows central airway involvement with extension to the carina, which is atypical compared to the usual peripheral radiologic presentation.

Therapeutically, due to the rarity of LSRCC, there is no standardized treatment protocol. Platinum-based doublet chemotherapy remains the most commonly used approach in advanced disease. In our patient, a weekly regimen of paclitaxel and carboplatin (AUC2) was selected, considering renal impairment and frailty. Although the disease remained stable after three cycles, the clinical course was complicated by a severe respiratory infection with a multidrug-resistant organism, leading to death despite appropriate antimicrobial therapy. This outcome is consistent with the poor prognosis often reported in the literature, where median overall survival in metastatic LSRCC ranges from six to 12 months [2].

Molecular profiling for driver mutations such as EGFR, ALK, and ROS1 is essential in advanced lung adenocarcinomas. However, these mutations appear to be less frequent in LSRCC. Our case was negative for EGFR and ALK, which excluded targeted therapies. Nonetheless, rare cases of ALK-rearranged LSRCC have been documented, suggesting that comprehensive genomic testing may uncover actionable mutations in select patients [6,7].

## Conclusions

In summary, LSRCC is a rare but highly aggressive subtype of lung adenocarcinoma, with a distinct clinicopathological profile and poor clinical outcomes. This case underscores the importance of prompt histological and immunohistochemical evaluation, thorough exclusion of extrapulmonary primaries, and early initiation of treatment. Larger multicenter studies and molecular analyses are needed to better characterize the biology of this tumor and improve therapeutic strategies.
